# L-carnitine attenuates autophagic flux, apoptosis, and necroptosis in rats with dexamethasone-induced non-alcoholic steatohepatitis

**DOI:** 10.1186/s40360-024-00820-z

**Published:** 2024-12-30

**Authors:** Ahmed E. Amer, Hamdy A. Ghoneim, Rania R. Abdelaziz, George S.G. Shehatou, Ghada M. Suddek

**Affiliations:** 1 Department of Pharmacology and Toxicology, Faculty of Pharmacy, Mansoura University, Mansoura, 35516 Egypt; 2 Department of Pharmacology and Biochemistry, Faculty of Pharmacy, Delta University for Science and Technology, International Coastal Road, Gamasa City, Dakahliya 35712 Egypt

**Keywords:** L-carnitine, Dexamethasone, NASH, Autophagy, Apoptosis, Necroptosis

## Abstract

**Background:**

UpToDate, no drugs have been approved to treat nonalcoholic steatohepatitis, the advanced stage of the most prevalent liver disease, non-alcoholic fatty liver disease. The present study was conducted to explore the potential influences of L-carnitine on the pathomechanisms of hepatic injury that mediate progression to non-alcoholic steatohepatitis in dexamethasone-toxified rats.

**Methods:**

Male Wistar rats were allocated as follows: dexamethasone group, rats received dexamethasone (8 mg/kg/day, intraperitoneally) for 6 days; DEXA-LCAR300, DEXA-LCAR500, and DEXA-MET groups, rats administered L-carnitine (300 or 500 mg/kg/day, IP) or metformin (500 mg/kg/day, orally) one week prior to dexamethasone injection (8 mg/kg/day, IP) and other six days alongside dexamethasone administration. Two groups of age-matched normal rats received either the drug vehicle (the control group) or the higher dose of L-carnitine (the drug-control group). At the end of the experiment, sets of biochemical, histological, and immunohistochemical examinations were performed.

**Results:**

L-carnitine (mainly at the dose of 500 mg/kg/day) markedly abolished dexamethasone-induced alterations in glucose tolerance, hepatic histological features, and serum parameters of hepatic function and lipid profile. Moreover, it significantly ameliorated dexamethasone-induced elevations of hepatic oxidative stress, SREBP-1 and p-MLKL protein levels, and nuclear FOXO1, LC3, P62, and caspase-3 immunohistochemical expression. Furthermore, it markedly diminished dexamethasone-induced suppression of hepatic Akt phosphorylation and Bcl2 immunohistochemical expression. The effects of L-carnitine (500 mg/kg/day) were comparable to those of metformin in most assessments and better than its corresponding lower dose.

**Conclusion:**

These findings introduce L-carnitine as a potential protective drug that may mitigate the rate of disease progression in non-alcoholic fatty liver disease patients with early stages or those at the highest risks.

**Supplementary Information:**

The online version contains supplementary material available at 10.1186/s40360-024-00820-z.

## Background

The most prevalent chronic liver disease is non-alcoholic fatty liver disease (NAFLD). Its incidence is expected to upsurge in tandem with the epidemics of type 2 diabetes (T2D) and obesity. NAFLD begins with hepatic steatosis, considered the disease’s initial stage. Consequently, it can advance to nonalcoholic steatohepatitis (NASH). If left untreated, NASH will develop into cirrhosis, liver failure, or hepatocellular carcinoma [1, 2]. Even so, no drugs are now approved to treat NASH, and the development of innovative therapeutics has remained a major challenge in the era of NASH research [1].

Many studies linked metabolic dysfunction to the development and progression of NAFLD. Metabolic dysfunction includes obesity, T2DM, dyslipidemia, hypertension, and metabolic syndrome, with insulin resistance (IR) functioning as the common underlying pathophysiological feature [3]. A dysfunctional cellular response to the insulin hormone in insulin-reliant tissues, such as hepatocytes, adipocytes, and cardiomyocytes, is the hallmark of the complex pathological condition of IR [4]. It is established that the phosphoinositide-3-phosphate kinase (PI3K)/Protein kinase B (Akt) pathway is the chief pathway that controls the insulin actions in the different tissues [5, 6]. Akt phosphorylation by insulin promotes phosphorylation, inactivation, and nuclear exclusion of forkhead box O1 (FOXO1), an action that is suppressed under IR conditions. FOXO1 governs transcriptions of various genes, including apoptosis, autophagy, and cell cycle arrest genes [7, 8].

Dexamethasone (DEXA) is a potent synthetic glucocorticoid (GC) with a high affinity for GC receptors [9]. DEXA was utilized in various previous studies to induce IR in whole tissues of experimental animals [10–13]. Moreover, it was demonstrated that DEXA administration to male Wistar rats leads to the advancement of NAFLD [14].

L-carnitine (LCAR) (3-hydroxy-4-trimethylaminobutyrate) is a quaternary amine that is found in most animal species, a wide range of microorganisms, and some higher plants [15]. LCAR has been widely marketed to improve exercise performance, well-being enhancement, and weight reduction. However, no clinical data consistently supports these indications [16]. The FDA has approved oral and intravenous LCAR to treat primary and secondary deficiencies [16]. The major physiological function of LCAR is to transport short-, medium-, and long-chain free fatty acids as acyl-carnitine esters through cell compartments. This transfer of acyl groups is followed by oxidation through the Krebs cycle [15]. LCAR supplementation diminished weight gain, hepatic steatosis, fasting serum triglycerides (TGs), and oxidative stress in hereditary hypertriglyceridemic rats [17]. It was also reported that LCAR attenuated hepatic injury in acetaminophen-treated mice [18]. LCAR administration was shown to reduce metabolic alterations associated with fructose intoxication in rodents [19, 20]. Moreover, recent studies have shown that LCAR ameliorated liver injuries induced by bile duct ligation [21], cisplatin [22], atrazine [23], lead acetate [24], and methotrexate [25], via anti-apoptotic, anti-inflammatory, and antioxidant actions.

Based on these data, this work investigated the potential ameliorative effects of LCAR, in comparison to MET, on different forms of liver injury induced by the DEX model of NASH in rats.

## Materials and methods

### Experimental animals

Male Wistar rats (270–300 g, *n* = 36) were supplied by VACSERA (Cairo, Egypt). They were given a week to acclimate before the initiation of studies. Rats were housed with a 12-hour on/off light program at room temperature (~ 25 ^o^C) and were fed with a standard chow during the experiment.

The handling of animals in our study followed the ethical standards outlined by the Ethical Committee for Scientific Research of the Faculty of Pharmacy, Mansoura University in compliance with the guidelines of Laboratory Animal Care (NIH publication no. 85 − 23, revised 2011). Current work is a part of the first author’s Ph.D. protocol which obtained ethical approval from the Mansoura University Animal Care and Use Committee (MU-ACUC) under the code of PHARM.PhD.22.11.5.

### Drugs and chemicals

LCAR was used as the commercially available LCAR ampules (1 g/5 ml) from an Arab Company for Pharmaceutical & Medicinal Plants (MEPACO- MEDIFOOD, Cairo, Egypt). DEXA was acquired from Amiriya Pharmaceutical Industries (Alexandria, Egypt) as dexamethasone sodium phosphate ampules. MET was purchased from MinaPharm (10^Th^ of Ramadan city, Egypt) as Glucophage tablets. Sigma Co. (Saint Louis, USA) provided thiobarbituric acid. The remaining chemicals were of the highest analytical quality.

### Experimental design

The DEXA model, previously reported by Kumar VH, Im NN, Huilgol SV, Yendigeri SM, K N and Ch R [26], is widely used for the induction of IR and hepatic steatosis in rodents [14, 27, 28]. Briefly, rats were randomly distributed into six groups, each of 6 animals, as follows: the **DEXA** group, rats received 0.5% carboxy methyl cellulose (0.5% CMC, orally) for 13 days. They were also injected with DEXA (8 mg/kg/day, IP) on days 8 to 13; the **LCAR** group received LCAR (500 mg/kg/day, IP) for 13 consecutive days; the **DEXA-LCAR300** and **DEXA-LCAR500** groups, rats were given LCAR (300 and 500 mg/kg/day, respectively, IP) starting seven days before DEXA administration and sustained for six days concomitantly with DEXA administration; the **DEXA-MET** group, animals were administered MET (500 mg/kg /day, prepared in 0.5% CMC, orally) as positive control drug, starting seven days before DEX administration and sustained for six days alongside with DEXA administration. A group of age-matched normal rats that received 2.5 mL/kg/day of 0.5% CMC served as the **control** group. The experimentation was ended after six days of DEXA injection, as demonstrated previously [14, 27, 28]. The doses of LCAR [19, 29, 30] and MET [31–33] were chosen based on earlier studies.

### Blood glucose-related measures

Oral glucose tolerance tests (OGTT) were performed as previously described. On the 6th day of DEXA administration, rats underwent a Ten hour fast. The fasting blood glucose (FBG) was then assessed using a blood sample drawn from each rat’s cut tail tip (served as time 0 levels). Afterward, rats were given a glucose solution (2 g/kg, orally), and blood glucose levels were determined after 30, 60, 90, and 120 min [34]. CareSens^®^ blood glucose meter (i-Sens, Seoul, South Korea) was used for glucose measurement.

### Change in rats’ body weights

Initially, the body weights of animals were measured at the start of the experiment. Then, they were weighed every other day of DEXA administration to adapt doses of drugs. Animals were also weighed before scarification. Calculations were made to determine the differences between the initial and end weights of the animals in each group.

### Blood sampling, specimen collection, and homogenate preparation

Secobarbital (50 mg/kg) was used to induce anesthesia in all rats at the termination of the experiment [35]. Blood samples were then collected from the retro-orbital sinus to obtain serum. The rats were then euthanized by decapitation. To calculate the liver weight/body weight ratios, liver tissues were then extracted, cleaned with ice-cold phosphate-buffered saline, dried on filter sheets, and weighed. Each rat had a section of its left lobe liver fixed in 10% neutral buffered formalin for later histological and immunohistochemistry examination. The remaining hepatic tissue was immediately placed in a freezer at -80 ^o^C. 10% liver tissue homogenates were prepared using a mini portable homogenizer (Omni International, USA) in PBS (0.05 M, pH 7.4). To eliminate cell debris, intact cells, nuclei, erythrocytes, and mitochondria, the homogenate was centrifuged at 10,000 rpm for 20 min. Malondialdehyde (MDA), Akt, p-Akt, and Sterol regulatory element binding proteins (SREBP-1), were afterward measured in the supernatants.

### Biochemical assays of serum samples

Serum activities of aspartate aminotransferase (AST), alanine aminotransferase (ALT), and lactate dehydrogenase (LDH) were assessed using commercially available assay kits from Human Diagnostic, Germany (Catalog numbers 12211, 12212, and 12214, respectively). A commercially available kit from Vitro Scient, Egypt (Catalog number 1081) was used for the determination of total bilirubin concentration. Alkaline phosphatase (ALP) was determined using an assay kit purchased from Agappe Diagnostic, Switzerland (Catalog number 51401002). γ-Glutamyltransferase (GGT) was assessed utilizing an assay kit from Spectrum Diagnostics, Cairo (Catalog number 247002). Spinreact, Spain, supplied albumin, triglycerides, total cholesterol, and high-density lipoprotein cholesterol (HDL-C) assay kits (Catalog numbers 1001020, 1001310, 1001090, and 1001095, respectively). The manufacturer’s instructions were followed for each assay. Non-HDL cholesterol concentrations which reflect the cholesterol contents of all atherogenic lipoproteins were determined as previously reported by Islam et al. 2022 [36] by subtracting HDL-C concentrations from the total cholesterol concentrations **(non-HDL = Total cholesterol - HDL-C).**

### Assay of hepatic oxidative stress

The degree of lipid peroxidation (measured as MDA) was used to assess the hepatic oxidative stress, following the well-established method of Ohkawa H, Ohishi N and Yagi K [37]. Briefly, thiobarbituric acid reacted with the MDA content of the hepatic homogenate in an acidic medium. After heating for 1 h at 95 °C, a pink color was produced proportional to the homogenate’s MDA concentration, which was measured colorimetrically at 532 nm.

### Enzyme-linked immunosorbent assay (ELISA)

Bradford method [38] was utilized for protein content determination in the hepatic issues utilizing a protein estimation kit (Genei, India). Phosph-Akt (pSer473)/pan-Akt ELISA kit (Catalog number RAB0012, Sigma Aldrich, USA) was used to assess hepatic concentrations of pan-Akt and phospho-Akt in hepatic homogenates according to the supplier’s instruction. p-Akt/Akt ratios were then calculated. Moreover, hepatic SREBP-1 was assayed using a commercially available ELISA kit (Catalog number E1312Ra, Bioassay Technology Laboratory, China).

### Western blot assay

Western blot technique was applied as reported previously [39]. The primary antibody against p-MLKL supplied by Invitrogen (MA, USA, cat. number PA5-105678) was utilized for the determination of hepatic p-MLKL protein concentration. Generated bands were quantified using the ImageJ program (National Institutes of Health, USA) relative to β-actin as a control protein.

### Histopathological examination and immunohistochemistry (IHC)

Formalin-fixed paraffin-embedded sections of livers were stained by H&E stain for examination of histopathological alterations. Pathological alterations have been scored as reported previously [40], with slight modification. For each rat section, the score value of each lesion was examined to obtain a total score for hepatic tissue that ranged from 0 to 12 (Supplementary Table S1).

IHC detection of hepatic microtubule-associated protein 1 A/1B light chain-3 (LC3) and P62 were performed utilizing 1^ry^ antibodies supplied by Servicebio Technology Co. (Wuhan, China, Catalog numbers GB13431 and GB11531, respectively). Moreover, IHC determination of the hepatic expression of caspase-3 and B-cell lymphoma 2 (BCL2) was conducted employing the 1^ry^ antibodies from ABclonal (MA, USA, Catalog numbers A11953 and A16776, respectively). IHC visualization of nuclear FOXO1 expression was performed using a primary antibody from cell signaling, USA (Catalog number #2880). Acquired images of IHC-stained hepatic tissues were imported to ImageJ application (ImageJ2, National Institutes of Health, USA) to quantify the percentage area of expression. A qualified pathologist who was fully blinded to the experimental data conducted the histopathological and IHC examinations.

### Statistical analysis

GraphPad Prism software (V 8.0.2, CA, USA) was utilized to perform the statistical analysis in this study. Data of histopathological scoring was analyzed by the Kruskal-Wallis test and demonstrated as medians ± interquartile ranges. Two-way analysis of variance (ANOVA) was employed to analyze OGTT records. One-way ANOVA followed by Tukey’s multiple comparisons was applied to analyze the remaining data, which presented as means ± standard deviations (SD). Statistical significance was considered at *P* < 0.05.

## Results

### Blood glucose-related parameters

**Fig. 1:**
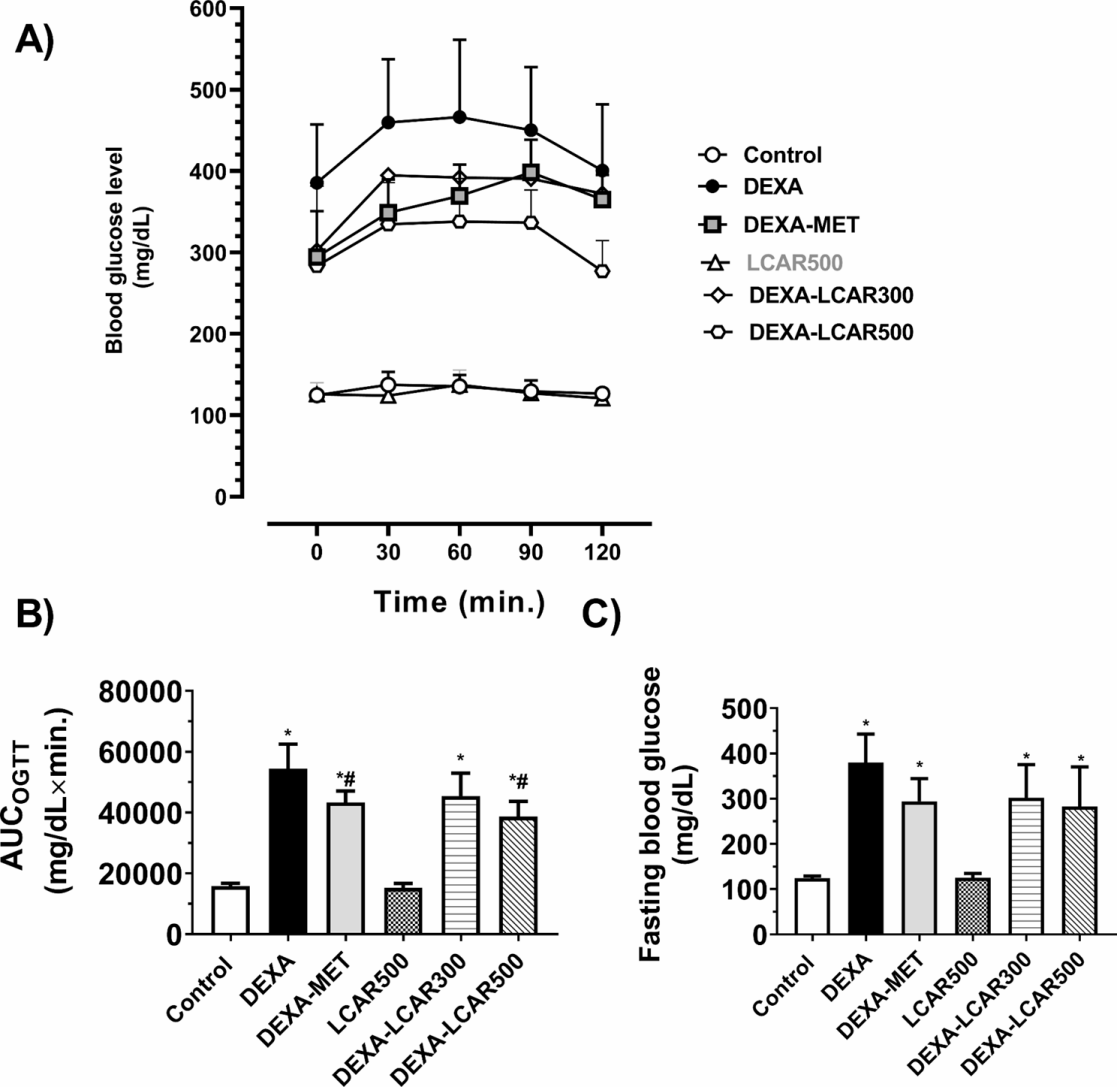
Effect of L-carnitine (LCAR, 300 and 500 mg/kg/day, IP) on glucose tolerance and fasting blood glucose in dexamethasone (DEXA)-toxified rats. **A** OGTT curves, **B** AUC_OGTT_, **C** FBG. Data are shown as means ± SD, *n* = 6 per group ^*, #^*P* < 0.05 versus control and DEXA groups, respectively. OGTT, oral glucose tolerance test; AUC: area under the curve; FBG, fasting blood glucose

OGTT curves are depicted in Fig. 1A. The DEXA group showed significant elevations in the area under the OGTT curve (AUC_OGTT_, by 3.4-fold, *P* < 0.0001)) and FBG (by 3.1-fold, *P* < 0.0001) relative to the control group (Fig. 1B and C).

Compared to the DEXA group, the AUC_OGTT_ in the DEXA-MET group was significantly reduced by 20.3% (*P* < 0.05). However, no significant change in FBG was observed (Fig. 1B and C).

DEXA-LCAR500 rats, but not the DEXA-LCAR300 group, demonstrated a marked decrease in AUC_OGTT_ by 28.9% relative to the DEXA group. Rats from DEXA-LCAR300 and DEXA-LCAR500 groups showed no marked difference in their FBG compared to those of the DEXA group. FBG And AUC_OGTT_ values from the DEXA-LCAR500 group were statistically comparable to those from the DEXA-MET group (Fig. 1B and C).

### General characteristics and serum liver function markers

The injection of DEXA into animals led to a considerable decrease in their body weights (*P* < 0.0001) compared to those of the control group. In addition, the liver indices of DEXA rats were 1.8 times significantly higher (*P* < 0.0001) than those of control rats (Table 1).

Although rats in the DEXA-MET group elicited considerably more weight loss than rats in the DEXA group, their liver indexes were significantly lesser by 24.8% (*P* < 0.0001, Table 1).

Administration of LCAR (300 and 500 mg/kg/day) didn’t markedly improve DEXA-induced weight loss. However, rats from DEXA-LCAR300 and DEXA-LCAR500 groups exhibited significantly lower liver indices by 13.2% (*P* < 0.05) and 14.0% (*P* < 0 0.01), respectively, compared to those of the DEXA group. Rats of the DEXA-MET group showed marked decreases in their body weights compared to those of either DEXA-LCAR300 or DEXA-LCAR500, while their liver indices were significantly lower than those of DEXA-LCAR300 group (Table 1).

DEXA administration significantly increased serum levels of ALT (5.5-fold), AST (1.8-fold), GGT (3.8-fold), ALP (6.2-fold), LDH (3.1-fold), and total bilirubin (of 2.7-fold) relative to the control rats (*P* < 0.0001, for all parameters). Moreover, serum albumin levels in DEXA rats were significantly lower (by 29.5%, *P* < 0.0001) than in the control group (Table 1).

Relative to the DEXA group, the DEXA-MET group showed considerable reductions in serum levels of LDH (35.6%, *P* < 0.01). Additionally, MET treatment significantly attenuated serum GGT, ALP, total bilirubin, and albumin to near-normal levels of the control group. In comparison to levels in the DEXA group, no significant changes in ALT or AST levels were seen in the DEXA-MET group (Table 1).

Administration of LCAR (500 mg/kg/day) to the DEXA rats resulted in a marked decrease in serum ALT (by 50.0%, *P* < 0.001), AST (by 25.7%, *P* < 0.05), and LDH (36.6%, *P* < 0.001) compared to the DEXA group. Moreover, administration of LCAR (300 and 500 mg/kg/day) significantly ameliorated DEXA-induced changes of serum GGT, ALP, total bilirubin, and albumin (*P* > 0.05 relative to the control group). These Effects were statistically comparable to those brought about by MET (Table 1).

**Table 1:** Effect of L-carnitine (LCAR, 300 and 500 mg/kg/day, IP) on general characteristics and serum hepatic function parameters in dexamethasone (DEXA)-toxified rats

	Control	DEXA	DEXA-MET	LCAR500	DEXA-LCAR300	DEXA-LCAR500
BW change (g)	3.50 ± 3.62	-82.00 ± 7.72^*^	-99.40 ± 10.71^*#^	4.83 ± 3.66	-77.67 ± 6.62^*$^	-83.00 ± 7.04^*$^
Liver index (%)	3.38 ± 0.04	6.22 ± 0.51^*^	4.68 ± 0.28^*#^	3.04 ± 0.14	5.40 ± 0.48^*#$^	5.35 ± 0.59^*#^
ALT (U/L)	55.00 ± 11.47	304.50 ± 91.90^*^	226.10 ± 19.63^*^	43.83 ± 12.54	222.00 ± 78.45^*^	152.30 ± 42.73^*#^
AST (U/L)	207.60 ± 20.66	379.30 ± 42.76^*^	325.40 ± 75.76^*^	157.70 ± 35.94	302.30 ± 69.95^*^	282.00 ± 44.20^#^
GGT (U/L)	18.83 ± 5.15	71.80 ± 12.25^*^	29.63 ± 6.28 ^#^	16.22 ± 3.66	35.00 ± 11.05^#^	34.33 ± 13.41^#^
ALP (U/L)	547.30 ± 95.72	3381 ± 1062^*^	852.20 ± 214.2^#^	599.30 ± 165.50	1103 ± 251.4^#^	416.00 ± 49.82^#^
LDH (U/L)	1742 ± 517.40	5480 ± 1199^*^	3529 ± 1139^*#^	1623 ± 115	5331 ± 645.50^*$^	3477 ± 745.90^*#!^
Albumin (g/dL)	4.55 ± 0.22	3.21 ± 0.35 ^*^	4.24 ± 0.47^#^	4.68 ± 0.21	4.19 ± 0.26^#^	4.33 ± 0.25^#^
Total bilirubin (mg/dL)	0.35 ± 0.05	0.96 ± 0.16^*^	0.37 ± 0.12^#^	0.19 ± 0.02	0.31 ± 0.15^#^	0.32 ± 0.08^#^

Data are shown as means ± SD, *n* = 6 per group

BW, body weight; AST, aspartate aminotransferase; ALT, alanine aminotransferase; GGT, gamma-glutamyl transferase; ALP, alkaline phosphatase; LDH, lactate dehydrogenase

^*,#,$,!^*P* < 0.05 versus control, DEXA, DEXA-MET, and DEXA-LCAR300 groups, respectively

### Lipid profile

In comparison to the control group, the DEXA rats exhibited marked elevations in serum total cholesterol (of 1.9-fold, *P* < 0.0001), serum non-HDL cholesterol (of 4.2-fold, *P* < 0.0001), and TGs (by 4.0-folds, *P* < 0.001). In contrast, serum HDL-C levels were significantly reduced by 36.0% in the DEXA group (*P* < 0 0.01 versus the control group).

Administration of MET or LCAR (300 or 500 mg/kg/day) to DEXA rats significantly ameliorated the DEXA-induced increase of serum TGs and reduction in the serum HDL-C (*P* > 0.05 relative to the control group). DEXA rats treated with MET or LCAR (300 or 500 mg/kg/day) demonstrated no significant difference in their serum total cholesterol levels relative to those of the DEXA group. However, they exhibited lower non-HDL cholesterol by 24.7% (*P* < 0.005), 43.6% (*P* < 0.0001), and 43.7% (*P* < 0.0001), respectively, compared to DEXA rats. The lipid profiles for all the study groups are presented in Table 2.

**Table 2:** Effect of L-carnitine (LCAR, 300 and 500 mg/kg/day, IP) on serum lipid profile in dexamethasone (DEXA)-toxified rats

	Control	DEXA	DEXA-MET	LCAR500	DEXA-LCAR300	DEXA-LCAR500
Total cholesterol (mg/dL)	101.60 ± 7.55	195.00 ± 31.70^*^	184.40 ± 41.20^*^	112.00 ± 19.28	151.20 ± 17.33^*^	154.00 ± 27.03^*^
HDL-C (mg/dL)	65.17 ± 5.38	41.71 ± 5.02^*^	69.50 ± 15.77^#^	64.00 ± 16.97	65.00 ± 9.21^#^	68.00 ± 6.93^#^
Non-HDL-C (mg/dL)	36.33 ± 6.77	152.70 ± 32.71^*^	115.00 ± 27.60^*#^	47.50 ± 2.88	86.17 ± 12.53^*#^	86.00 ± 20.34^*#^
TGs (mg/dL)	98.33 ± 19.16	396.50 ± 212.8^*^	211.00 ± 63.78^#^	113.80 ± 57.27	200.80 ± 80.24^#^	161.00 ± 40.58^#^

Data are shown as means ± SD, *n* = 6 per group

HDL-C, high-density lipoprotein cholesterol; TGs, triglycerides

^*, #^*P* < 0.05 versus control and DEXA groups, respectively

### Liver morphology and histopathological changes

**Fig. 2:**
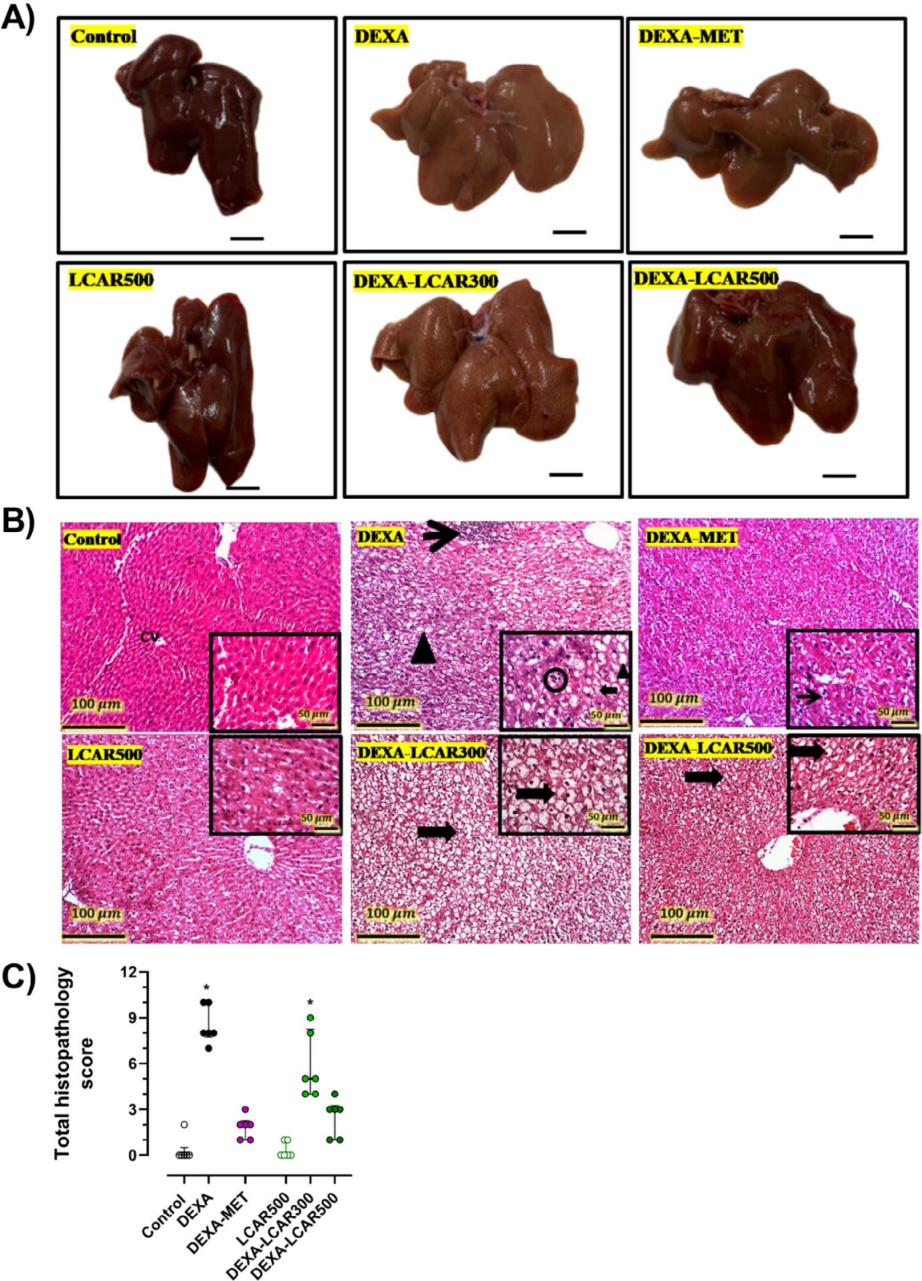
Effect of L-carnitine (LCAR, 300 and 500 mg/kg/day, IP) on liver morphology and hepatic histopathological features in dexamethasone (DEXA)-toxified rats. **A** Representative macroscopic images of the livers from the different groups (scale bar, 1 cm), **B** Histological microimages of H&E-stained hepatic Sect. (100× magnification; scale bar, 100 μm and 400× magnification in insets; scale bar, 50 μm), **C** Semi-quantitative scoring of the hepatic histopathological alterations. **Panel B.** Control and LCAR500 groups display normal hepatic cords around central veins. DEXA-group shows steatohepatitis with inflammatory foci (thin arrow) and diffused hepatic vacuolation (arrowhead). Inset, numerous macro (arrowhead) and micro lipid droplets (thick arrow) with few invading neutrophils (circle). DEXA-MET groups show haptic sections with moderately swelled hepatocytes (thin arrow). The section of the DEXA-LCAR300 group demonstrates diffuse severe ballooned hepatocytes (thick arrow), while the section from the DEXA-LCAR500 group displays modest ballooning degeneration of hepatocytes (thick arrow). **Panel C.** Data are shown as medians ± interquartile range, *n* = 6 per group; * *P* < 0.05 versus the control group

Macroscopic images of liver tissue in the study groups are shown in Fig. 2A. Livers from the control, LCAR500, DEXA-MET, and DEXA-LCAR500 groups showed normal color and morphologic appearance. However, livers from DEXA and DEXA-LCAR300 groups show a yellowish steatotic appearance. Representative H&E-stained hepatic sections are presented in Fig. 2B. The control and LCAR500 groups showed normal hepatic cords around central veins. Conversely, hepatic sections from the DEXA group showed lobular steatohepatitis, focal inflammatory infiltration, diffused macro and micro vesiculation, and ballooning degeneration. Moreover, mild lobular fibrosis and eosinophilic intracellular inclusions (Mallory Denk bodies) were found in some sections. Hepatic sections from the DEXA-MET group showed moderately expanded hepatocytes. Sections from the DEXA-LCAR300 group showed diffuse severe ballooned hepatocytes. Moreover, congested, and dilated portal veins, mild periportal fibrosis, some lymphocytes, and Denk Mallory bodies were observed in some sections. Liver tissues from the DEXA-LCAR500 group displayed moderate ballooning degeneration of hepatocytes.

Histopathological scoring revealed that DEXA-administrated rats exhibited pathological scores that were markedly higher than the control group (Fig. 2C). Conversely, hepatic tissues from the DEXA-MET and the DEXA-LCAR500 groups demonstrated lower scores that were insignificant from those of the control group. However, rats from the DEXA-LCAR300 group showed scores that were significantly higher than the control group (Fig. 2C).

### Hepatic MDA content, p-Akt/Akt ratios, nuclear FOXO1 expression, and SREBP1

**Fig. 3:**
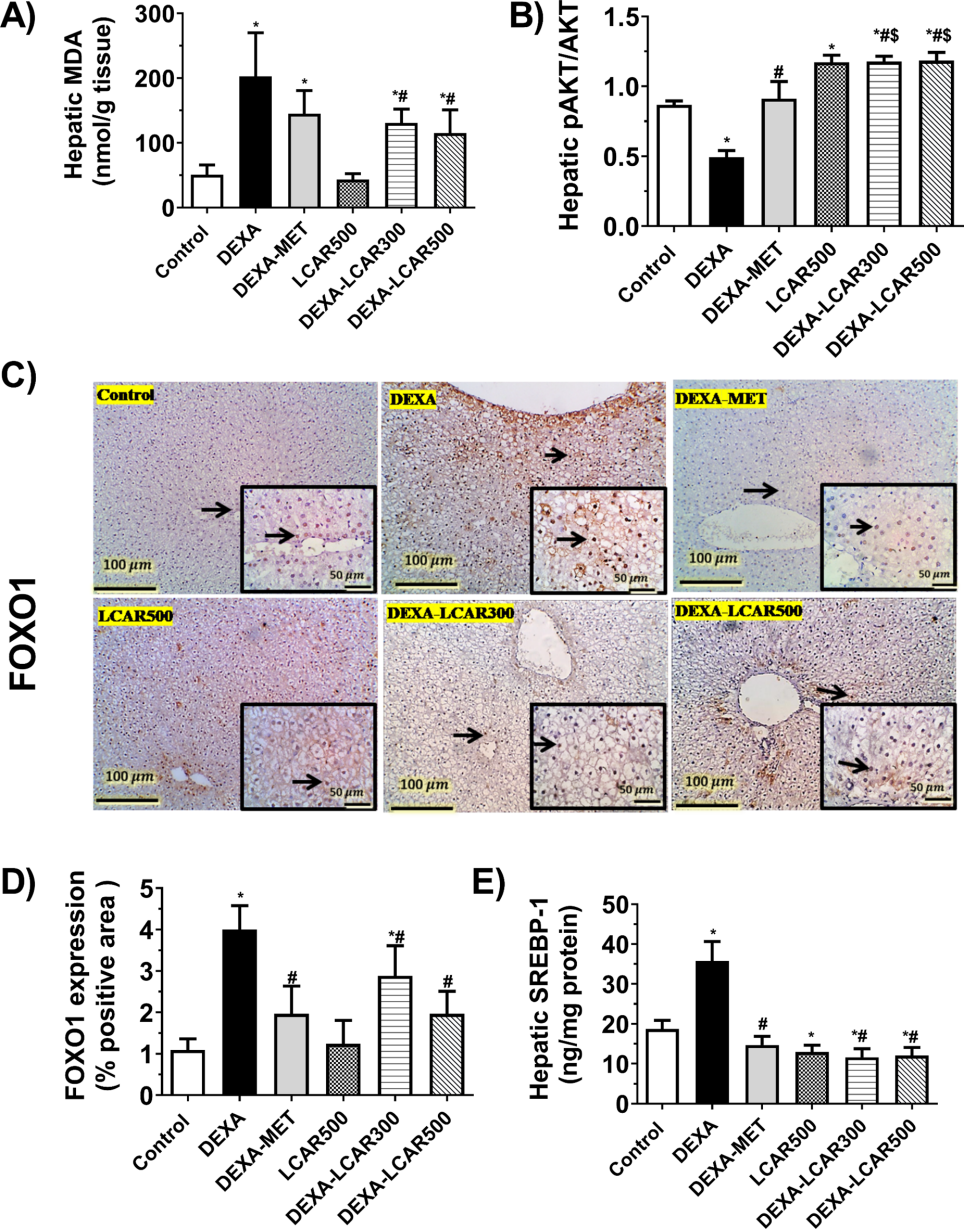
Effect of L-carnitine (LCAR, 300 and 500 mg/kg/day, IP) on hepatic oxidative stress, Akt phosphorylation, FOXO1 nuclear immunoexpression, and SREBP1 protein levels in dexamethasone (DEXA)-toxified rats. **A** Hepatic MDA concentrations, **B** Hepatic ratios of p-Akt/Akt, **C** Representative IHC-stained sections against FOXO1 in the study groups (100× magnification; scale bar, 100 μm and 400× magnification in insets; scale bar, 50 μm). Arrows refer to positive nuclear expression, **D** %areas of nuclear IHC experssion of FOXO1, **E** Hepatic protein level of SREBP1. **Panels A**,** B**,** D and E**: Data are shown as means ± SD, *n* = 6 per group; ^*,#^*P* < 0.05 versus control and DEXA groups, respectively. IHC, immunohistochemistry; MDA, malondialdehyde; FOXO1, forkhead box protein O1; SREBP1, sterol regulatory element binding proteins

Administration of DEXA resulted in a significant increase in hepatic MDA contents by 4-fold (*P* < 0.0001) compared to the control group (Fig. 3A). The DEXA-MET rats showed hepatic MDA contents, which were not markedly changed from those of the DEXA group (Fig. 3A).

Treating DEXA rats with LCAR (300 and 500 mg/kg/day) considerably mitigated DEXA-induced increase of hepatic MDA content by 35.5% (*P* < 0.05) and 43.4% (*P* < 0.01), respectively, relative to the untreated DEXA group (Fig. 3A).

Rats from the DEXA group showed markedly reduced hepatic p-Akt/Akt ratios by 43.0% (*P* < 0.0001) compared to the control group (Fig. 3B). Administration of MET to the DEXA rats markedly ameliorated hepatic p-Akt/Akt ratios to values that were statistically comparable to those of the control group (Fig. 3B). Treating DEXA rats with LCAR 300 or 500 mg/kg/day markedly encouraged their hepatic p-Akt/Akt ratios by 2.38 and 2.39-fold (*P* < 0.0001), respectively, relative to rats of the DEXA group. Hepatic p-Akt/Akt ratios of DEXA-LCAR500 rats were considerably greater (*P* < 0.0001) than those from the DEXA-MET group (Fig. 3B).

Hepatic sections from the control, LCAR500, and DEXA-MET groups demonstrated negative to limited faint FOXO1 nuclear expressions. Contrariwise, DEXA group sections elicited diffuse nuclear overexpression of FOXO1 in hepatocytes. Hepatic sections from the DEXA-LCAR300 group exposed mild to modest expression of FOXO1 in the nucleus of different inflammatory cells, while liver sections of the DEXA-LCAR500 group exhibited mild nuclear expression (Fig. 3C). The percentage area of FOXO1 nuclear expression in the study groups is presented in Fig. 3D.

Rats from the DEXA group elicited significantly higher hepatic SREBP1 levels (by 1.9-fold, *P* < 0.0001) relative to those from the control group (Fig. 3E). Administration of MET to DEXA rats markedly suppressed the hepatic SREBP1 expression by 58.9% (*P* < 0.0001) compared to the DEXA group (Fig. 3E). Treating DEXA rats with LCAR (300 and 500 mg/kg/day) resulted in a significant decrease in their hepatic SREBP1 levels by 67.6% and 66.2% (*P* < 0.0001), respectively, compared to the DEXA rats (Fig. 3E).

### Hepatic expression of autophagic-related proteins LC3 and P62

**Fig. 4:**
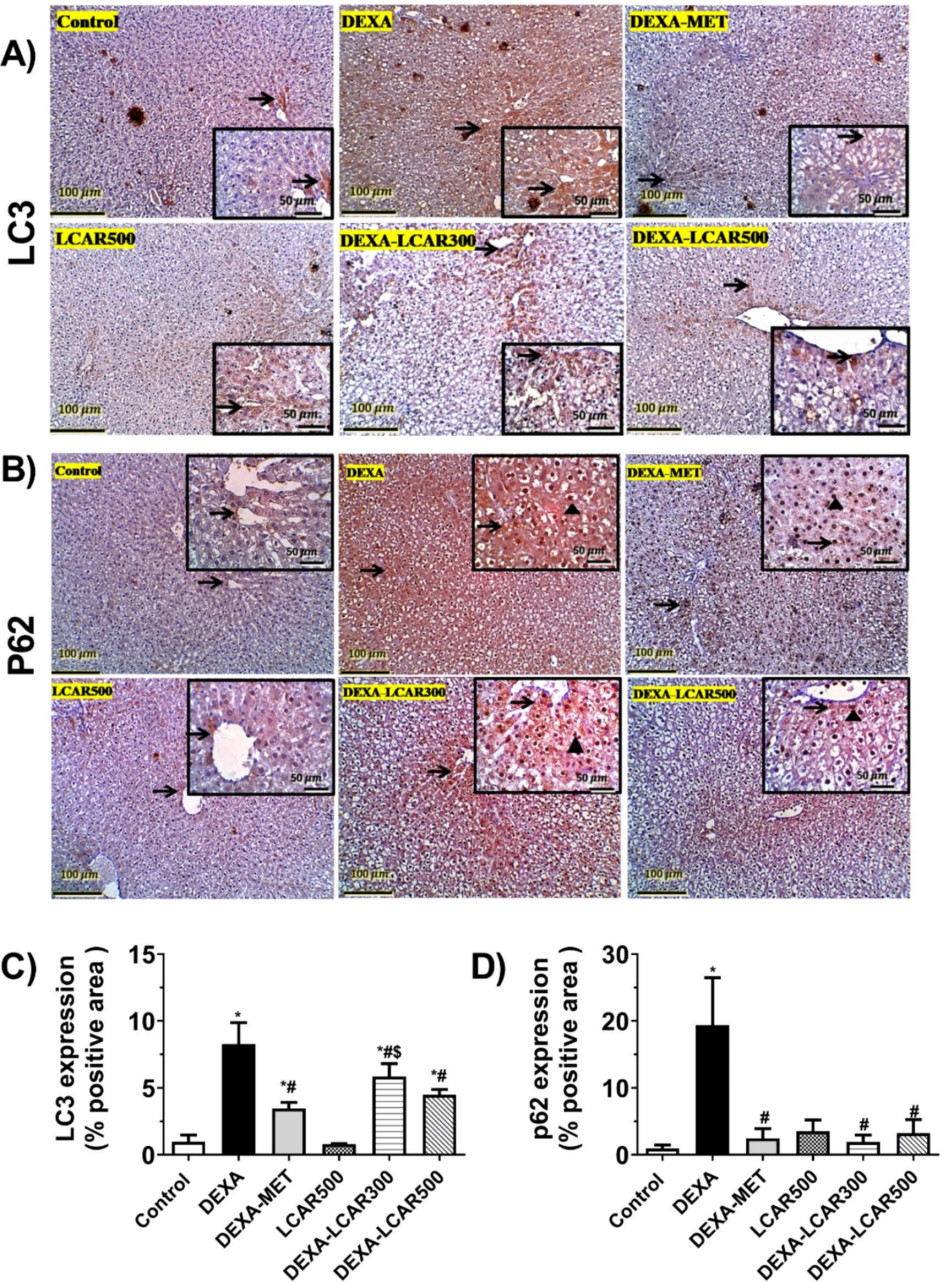
Effect of L-carnitine (LCAR, 300 and 500 mg/kg/day, IP) on the hepatic expression of autophagic-associated proteins LC3 and P62 in dexamethasone (DEXA)-toxified rats. **A and B.** Expressive images of immunostained liver sections against LC3 and P62, respectively (100× magnification; scale bar, 100 μm and 400× magnification in insets; scale bar, 50 μm). **C and D.** %areas of hepatic IHC expression of LC3 and P62, respectively. **Panel (A)** Insets display cytoplasmic LC3 expression; Thin arrows refer to positive expression. **Panel (B)** Insets display cytoplasmic and nuclear P62 immunostaining, thin arrows refer to positive cytoplasmic expression; Arrowheads refer to positive nuclear expression. **Panel C and D.** Data are shown as means ± SD, *n* = 6 per group; ^*,#,$^*P* < 0.05 versus control, DEXA, and DEXA-MET groups, respectively. LC3, microtubule-associated protein 1 A/1B light chain-3

Hepatic sections from control and LCAR500 rats exhibited mild cytoplasmic expression of LC3 in the hepatocytes. Conversely, DEXA-administrated rats showed diffuse strong cytoplasmic expression of LC3 in hepatocytes with steatosis. DEXA rats treated by MET or LCAR (300 and 500 mg/kg/day) demonstrated moderate cytoplasmic LC3 expression in their hepatocytes (Fig. 4A).

On the other hand, examination of hepatic P62 immunoexpression revealed that hepatic sections from the control, LCAR500, DEXA-MET, DEXA-LCAR300, and DEXA-LCAR500 groups exhibited faint to mild cytoplasmic expression along with negative or limited nuclear expressions. Contrariwise, rats from the DEXA group displayed strong significant cytoplasmic and nuclear immunostaining in their hepatocytes (Fig. 4B).

Percentages of positive LC3 and P62 immunostaining in the study groups are presented in Fig. 4C and D, respectively.

### Hepatic expression of apoptotic-associated proteins caspase-3 and Bcl2

**Fig. 5:**
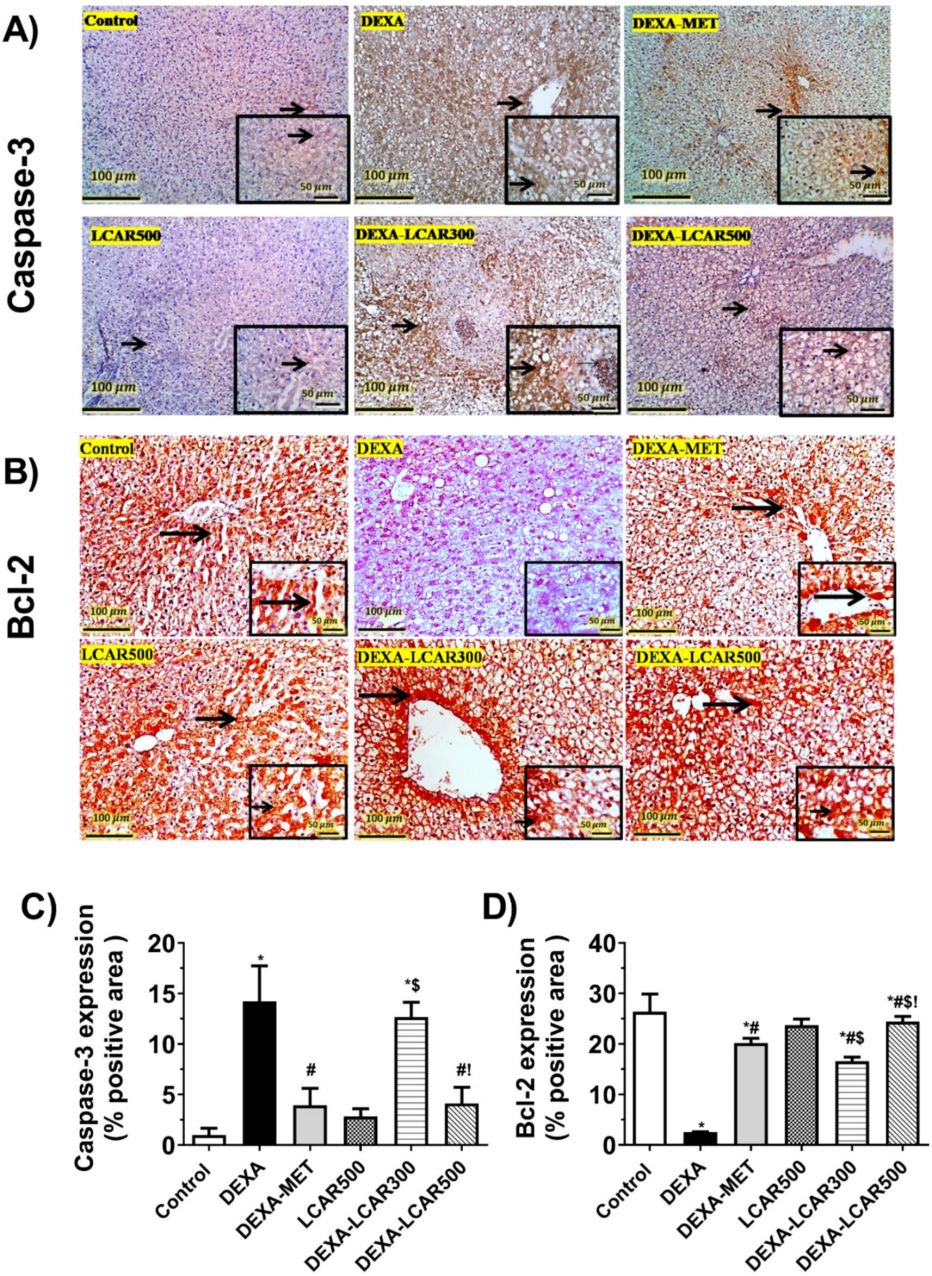
Effect of L-carnitine (LCAR, 300 and 500 mg/kg/day, IP) on the hepatic expression of apoptotic-associated proteins caspase-3 and BCL2 in dexamethasone (DEXA)-toxified rats. **A and B.** Expressive images of IHC staining of liver sections against caspase-3 and BCl2, respectively (100× magnification; scale bar, 100 μm and 400× magnification in insets; scale bar, 50 μm). **C and D.** %areas of positive immunoexpression of caspase-3 and BCl2, respectively. **Panel A and B.** Insets display caspase3 and Bcl-2 immunostaining, respectively. Arrows refer to positive expressions. High power in the DEXA-LCAR300 group shows nuclear expression in invading neutrophils and lymphocytes (Thin arrow). **Panel C and D.** Data are shown as means ± SD, *n* = 6 per group; ^*,#,$,!^*P* < 0.05 versus control, DEXA, DEXA-MET, and DEXA-LCAR300, respectively. BCl2, B-cell lymphoma-2

Representative IHC expressions of hepatic caspase-3 and BCL2 in the study groups are presented in Fig. 5A and B, respectively.

Hepatic sections from control and LCAR500 showed faint cytoplasmic expression of caspase-3 and markedly high expression of Bcl2 in the hepatocytes. Conversely, liver sections of the DEXA group showed diffuse strong cytoplasmic expression of caspase-3 in the hepatocytes with macrovesicular steatosis and minimal faint expression of BCL2.

The DEXA-LCAR300 rats showed hepatic sections with strong cytoplasmic caspase-3 expression in their vacuolated hepatocytes, while they displayed weak to moderated expression of BCL2. However, hepatic tissues from rats of the DEXA-MET and DEXA-LCAR500 groups exhibited mild to moderate cytoplasmic expression of caspase-3, while they demonstrated strong BCL2 expression.

Percentage areas of immunoexpression of caspase-3 and BCL2 in the study groups are presented in Fig. 5C and D, respectively.

### Hepatic expression of the necroptotic marker p-MLKL

**Fig. 6:**
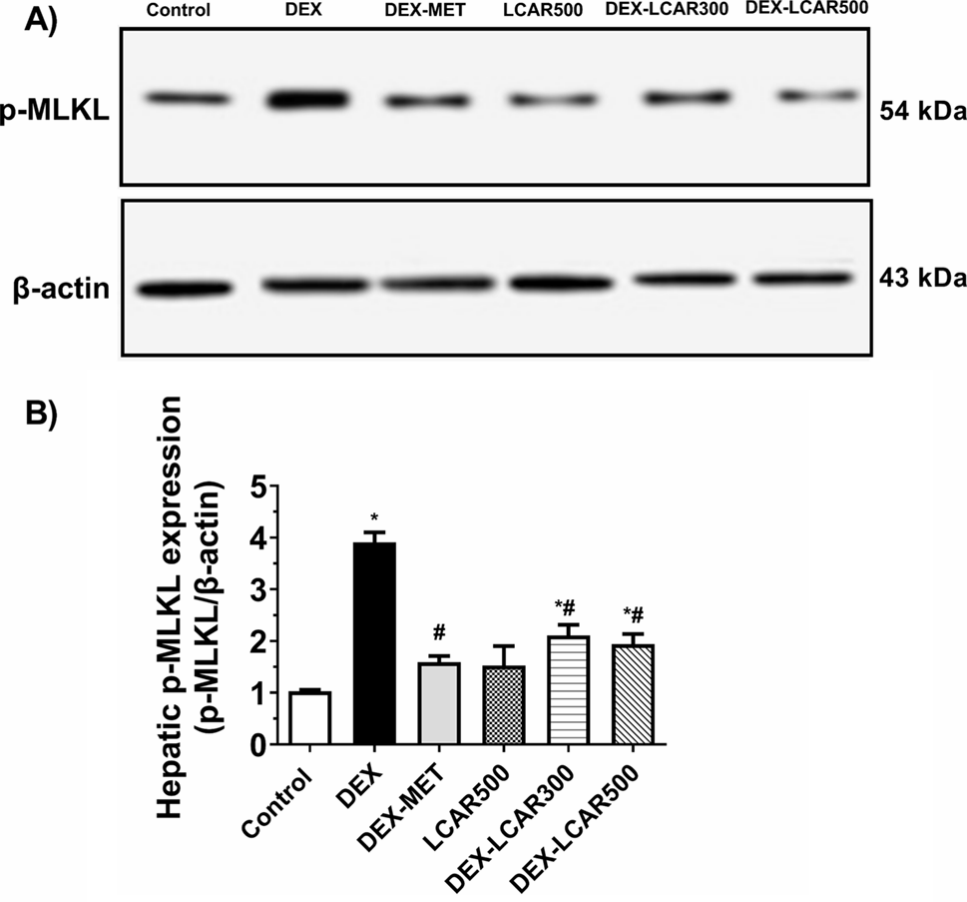
Effect of L-carnitine (LCAR, 300 and 500 mg/kg/day, IP) on the hepatic protein expression of the necroptotic-associated protein p-MLKL in dexamethasone (DEXA)-toxified rats. **A** Expressive western blots of hepatic p-MLKL protein expression in the different groups, **B** Densitometric analyses of band intensities. Data are shown as means ± SD, *n* = 3/group; ^*, #^*P* < 0.05 versus control and DEXA groups, respectively. Uncropped western blots are shown in supplementary Fig. 1. MLKL, mixed lineage kinase domain-like

Expressive Western blot analysis of hepatic expression of p-MLKL in the study groups is presented in Fig. 6A.

Administration of DEXA to rats induced a marked increase of hepatic p-MLKL protein expression by 3.8-fold (*P* < 0.0001) relative to the control group (Fig. 6B). MET treatment significantly diminished the DEXA-induced increase in hepatic p-MLKL protein (*P* > 0.05 vs. control rats, Fig. 6B). Moreover, DEXA rats treated by LCAR (300 and 500 mg/kg/day) exhibited reduced hepatic p-MLKL protein expression by 46.1% and 50.4%, respectively, relative to the untreated DEXA groups (*P* < 0.0001, Fig. 6B).

## Discussion

IR has a central role in the development and progression of NAFLD. DEXA administration was shown to stimulate the development of IR [10–13] and NAFLD in numerous reports [9, 14, 41]. The ability of DEXA to induce these pathological changes in a relatively short period serves as an additional advantage behind its selection [26, 42, 43]. In this work, DEXA administration induced pathological features of NASH accompanied by dysregulation of hepatic function serum markers and several metabolic abnormalities, including hyperglycemia, hypercholesterolemia, and hypertriglyceridemia. Interestingly, LCAR treatment (mainly at a dose of 500 mg/kg/day) significantly attenuated these injurious actions of DEXA.

MET was selected as a positive control drug in this work based on several studies demonstrating its hepatoprotective actions in NAFLD/NASH patients. Moreover, its ability to mitigate hepatic steatosis was reported in various experimental studies [44–46], which was attributed to the induction of autophagy [47, 48]. Furthermore, it has been reported that MET abolished palmitate-induced lipotoxicity and hepatic apoptosis [49]. Most recently, MET has been shown to attenuate hepatic steatosis by autophagy and necroptosis modulation [50]. Supporting these notions, MET successfully diminished DEXA-induced pathological alterations and dysregulations of different parameters of hepatic apoptosis, autophagy, and necroptosis in the current study. Remarkably, the higher dose of our investigated drug LCAR (500 mg/kg/day), excreted effects on these aspects of hepatic injury in DEXA-injected rats that were comparable to those of MET.

The PI3K/Akt pathway is the major insulin pathway that controls the insulin actions in the body [5, 6]. Results of this work show that DEXA suppressed Akt phosphorylation, which agrees with previous experimental studies [51–53]. One of the major downstream effectors of Akt is FOXO1 which is phosphorylated and nuclear excluded in response to Akt activation [7]. FOXO1 governs transcription of a wide range of genes, including apoptosis and autophagy genes. Moreover, it controls the transcription of glucose-6-phosphatase, a master regulator of gluconeogenesis [54]. Several studies have shown that excessive activity of FOXO1 in mice can lead to hepatosteatosis [55–60]. Additionally, in cirrhotic patients, FOXO1 levels are strongly correlated with the level of hepatic damage [61]. DEXA-administrated rats exhibit high nuclear expression of FOXO1, which could be a consequence of Akt deactivation. LCAR treatment significantly increased the p-Akt/Akt ratio along with suppression of FOXO1 nuclear expression. The ability of LCAR to activate Akt was reported previously [62].

Oxidative stress was shown to stimulate FOXO1 nuclear transition [62]. DEXA administration increased hepatic oxidative stress, as indicated by the enhancement of hepatic MDA contents in DEXA-administrated rats. However, LCAR treatment significantly reduced hepatic MDA levels. LCAR showed antioxidant actions in animals with liver fibrosis induced by bile duct ligation [21]. Moreover, a recent study reported the ability of LCAR to reduce oxidative stress in atrazine-induced hepatotoxicity [23]. Collectively, the ability of L-CAR to activate Akt, in addition to its antioxidant actions, may explain its attenuating effects on hepatic FOXO1 expression.

Additionally, our findings showed that DEXA administration significantly increased hepatic SREBP-1 protein levels. SREBP-1 is a transcription factor that plays a critical role in *de novo* lipogenesis (DNL), which is often induced in cases of NAFLD and in response to conditions such as endoplasmic reticulum (ER) stress, hyperglycemia, and oxidative stress [63–65]. SREBP-1 governs the expression of the main genes of DNL and TGs synthesis [66]. Previous studies have linked adenovirus-mediated overexpression or activation of FOXO1 to induced SREBP1 expression and transcriptional activity, leading to the accumulation of lipids in hepatic tissue [67, 68]. Additionally, it was shown that activated FOXO1 induced SREBP-1 and very low-density lipoprotein (VLDL) expression and promoted hypertriglyceridemia through modulation of microsomal TG transfer protein (MTP) [68]. Furthermore, a recent study has reported that SREBP1 impairs autophagic flux and leads to the development of hepatic steatosis in mice models fed with a high-fat diet (HFD) [69]. In the present study, LCAR significantly suppressed DEXA-induced elevation of SREBP-1. In agreement with our findings, LCAR was shown to suppress overexpression of SREBP1c induced by HFD in obese diabetic mice [70].

The hepatic injury associated with NAFLD has been linked to several pathomechanisms, including apoptosis, autophagy dysfunction, and necroptosis [71–73]. Notably, key markers for these injury mechanisms were induced upon DEXA administration.

FOXO1 is known to induce autophagy by activating autophagy-related genes [74, 75]. Autophagy is a cellular degradation process that helps promote cell survival during times of stress by providing energy and eliminating damaged organelles and proteins [76]. It has been reported that in the early stages of steatosis, autophagy is activated to fight against lipotoxicity. However, in persistent metabolic stress, autophagic flux is later blocked [71]. Autophagic flux is known to be blocked in both human and experimental settings of NAFLD [72, 76–78]. In the present study, hepatic sections from the DEXA group displayed high expression of LC3, a major protein of autophagosome, which suggests induction of autophagy. Additionally, P62 expression was induced following DEXA application. P62 is responsible for transferring organelles and polyubiquitinated proteins to the autophagosome-lysosome for degradation [79]. Therefore, both LC3 and P62 accumulations imply the obstruction of autophagic flux in DEXA-administered rats. It is noteworthy that the administration of LCAR was able to suppress the expression of P62, while the expression of LC3 remained higher than that of the control group in the current study. This suggests that the autophagy process was still initiated, while LCAR ameliorated the obstruction of autophagy flux induced by DEXA.

One of the consequences of P62 accumulation is the activation of apoptosis. Accumulated P62 can activate caspases [80]. Additionally, in various experimental settings, the induction of apoptosis has been observed as a consequence to the increase of nuclear expression of FOXO1 [81, 82]. Furthermore, oxidative stress is one of the most known inducing factors of hepatic apoptosis [83]. It is well-established that hepatocyte apoptosis is a key feature in NAFLD/NASH patients [71]. Herein, DEXA administration resulted in a marked increase of caspase 3, which is typically responsible for cell destruction during intrinsic and extrinsic apoptosis in human cells [80]. Moreover, DEXA administration markedly diminished hepatic Bcl-2 expression. Bcl-2 is one of the most known pro-survival anti-apoptosis proteins that directly binds and inhibits pro-apoptotic inducers [84]. LCAR abolished DEXA-induced accumulation of P62, increased nuclear FOXO1 expression, oxidative stress, and suppression of Bcl-2 expression, which collectively could explain the suppression of caspase-3 expression upon LCAR treatment. Supporting our findings, it has been shown that LCAR prevented lead acetate-induced hepatic apoptosis in rats via its antioxidant actions [24]. Moreover, LCAR prevented methotrexate-induced caspase-3 activation in isolated rat hepatocytes [25]. The anti-apoptotic effect of LCAR (500 mg/kg/day) treatment was higher than that of LCAR (300 mg/kg/day), which might explain the enhanced outcomes of the higher dose.

Cellular death is often caused by apoptosis, but in cases of extensive injury and increased levels of death signaling, the necroptotic pathway may also be activated to ensure cell death [85]. In this work, administration of DEXA increased the protein expression of P-MLKL, a downstream effector of necroptosis. When phosphorylated, MLKL moves to the plasma membrane and assembles into oligomers or polymers, which bind to negative-charged phospholipids. This results in the formation of pores that cause damage to the cell membrane, ultimately leading to cell death [73]. Previous research has shown that higher expression of MLKL inhibits autophagic flux in a diet model of NAFLD-NASH [73]. Recently, it has been reported that the active nuclear FOXO1 encourages the p-MLKL expression and necroptosis in NASH experimental model [86]. Additionally, FOXO1 inhibition has been found to ameliorate necroptosis and ER stress in mice fed with HFD [87]. Treatment with LCAR significantly reduced DEXA-induced elevation of p-MLKL expression, possibly due to its capability to suppress the metabolic stress and inhibit FOXO1 nuclear expression.

**Fig. 7:**
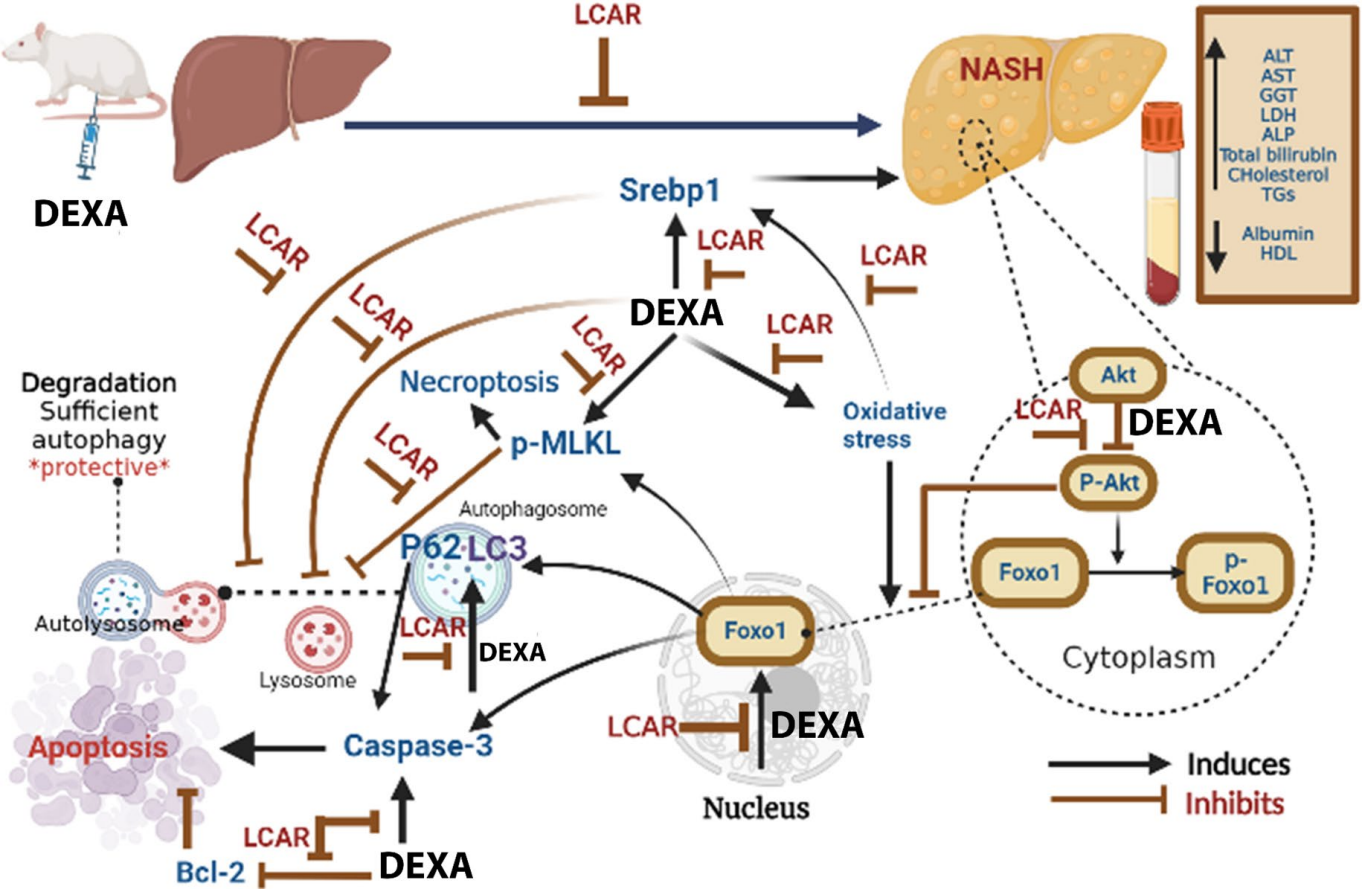
A graphical abstract showing the possible mechanisms that might underlie the ameliorating impacts of L-carnitine on hepatic injury in dexamethasone (DEXA)-toxified rats. DEXA, dexamethasone; LCAR, L-carnitine; NASH, nonalcoholic steatohepatitis; AST, aspartate aminotransferase; ALT, alanine aminotransferase; GGT, gamma glutamyl transferase; ALP, alkaline phosphatase; LDH, lactate dehydrogenase; HDL, high-density lipoprotein cholesterol; TGs, triglycerides; BCL2, B-cell lymphoma-2; MDA, malondialdehyde; LC3, microtubule-associated protein 1 A/1B light chain-3; MLKL, mixed lineage kinase domain-like; FOXO1, forkhead box protein O1; SREBP1, sterol regulatory element binding proteins

The major constraint of the current study is that we conducted a protective protocol for LCAR preceding the NASH induction. Therefore, it is unclear if it could attenuate the pre-existing NASH. Future studies that investigate the therapeutic potential of LCAR are therefore required.

In conclusion, LCAR (mainly at its higher investigated dose 500 mg/kg/day) markedly attenuated the injurious hepatic consequences induced by DEXA-toxification by various interrelating mechanisms (Fig. 7), including reduction of oxidative stress, enhancement of Akt activation, suppression of nuclear FOXO1 expression, diminishing the accumulation of autophagic molecules, inhibition of apoptosis effector caspase-3 alongside the stimulation of pro-survival protein Bcl-2, and abolishing the necroptotic p-MLKL protein expression. In our study, L-carnitine was primarily effective in its higher dose (500 mg/kg/day) indicating the necessity of dosing in the therapeutic effectiveness. studies remain to investigate the ability of LCAR to reduce disease progression in NAFLD/NASH patients and protect those at high risk for fatty liver diseases.

## Electronic supplementary material

Below is the link to the electronic supplementary material.

Supplementary Material 1

## Data Availability

No datasets were generated or analysed during the current study.

## Abbreviations

NAFLD: Nonalcoholic fatty liver disease
NASH: Nonalcoholic steatohepatitis
SREBP1: Sterol regulatory element binding proteins
FOXO1: Forkhead box protein O1
LC3: Microtubule-associated protein 1 A/1B light chain 3
Bcl-2: B-cell lymphoma
MLKL: Mixed lineage kinase domain-like
