# Supplementary material for: L-carnitine attenuates autophagic flux, apoptosis, and necroptosis in rats with dexamethasone-induced non-alcoholic steatohepatitis
Source: BMC Pharmacol Toxicol. 2024 Dec 30;25:102. doi: 10.1186/s40360-024-00820-z (PMC11684100; doi:10.1186/s40360-024-00820-z)
Supplement: Supplementary file 1 — Supplementary Material 1 [file 40360_2024_820_MOESM1_ESM.pdf]

Table S1: Scoring system for hepatic pathological alterations utilized in the current study

score	Steatosis	Ballooning degeneration	Inflammation	Fibrosis
0	None	None	None	None
1	Less than 5% of tissue affected	Minimal, few	1 foci per 12 fields of view	Periportal only or Perisinusoidal only
2	More than 5- 50% of tissue affected	Mild to moderate, multifocal to coalescing degenerated area	2-3 foci/12 fields exhibited inflammation	Perisinusoidal + periportal or p eriportal septa
3	More than 50% of tissue exhibited steatosis	Diffuse, many	More than 3 foci/12 fields exhibited inflammation	Bridging

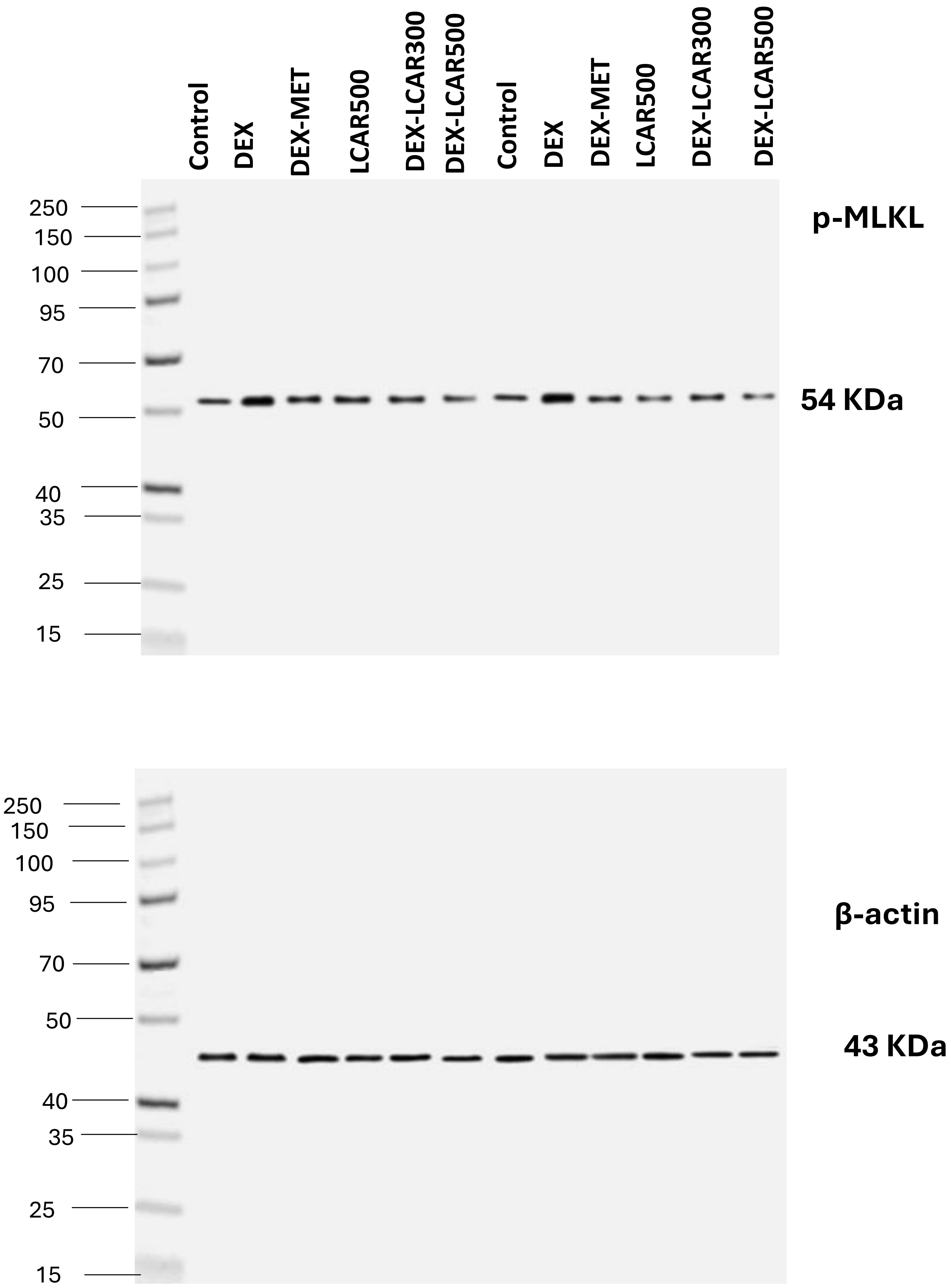

Control
DEX
DEX-MET
LCAR500
DEX-LCAR300
DEX-LCAR500
Control
DEX
DEX-MET
LCAR500
DEX-LCAR300
DEX-LCAR500
p-MLKL
250
150
100
95
70
50
40
35
25
15
54 KDa
β-actin
250
150
100
95
70
50
40
35
25
15
43 KDa
